# Awareness and perceptions on prevention, first aid and treatment of snakebites among Sri Lankan farmers: a knowledge practice mismatch?

**DOI:** 10.1186/1745-6673-9-20

**Published:** 2014-05-13

**Authors:** Anjana Silva, Faiz Marikar, Arumugam Murugananthan, Suneth Agampodi

**Affiliations:** 1 Department of Parasitology, Faculty of Medicine and Allied Sciences, Rajarata University of Sri Lanka, Saliyapura, 50008 Anuradhapura, Sri Lanka; 2 Department of Biochemistry, Faculty of Medicine and Allied Sciences, Rajarata University of Sri Lanka, Saliyapura, 50008 Anuradhapura, Sri Lanka; 3 Division of Parasitology, Department of Pathology, Faculty of Medicine, University of Jaffna, Jaffna, Sri Lanka; 4 Department of Community Medicine, Faculty of Medicine and Allied Sciences, Rajarata University of Sri Lanka, Saliyapura, 50008 Anuradhapura, Sri Lanka

**Keywords:** Snakebite, Farming, Prevention

## Abstract

**Background:**

Snakebite is a global health problem associated with high morbidity and mortality. In Sri Lanka, snakebite is mainly an occupational health hazard associated with farming. Understanding awareness and perceptions in risk populations on the preventive measures, first aid and treatment for snakebite becomes pivotal in designing snakebite prevention and control programs. Using an investigator assisted self completed questionnaire, we assessed the awareness and perceptions of 176 part-time and full-time, Chena and paddy farmers from three dry zone districts of Sri Lanka where agriculture is the main economic activity.

**Findings:**

High percentages of the participants were aware of practices that minimize snakebites in houses and outside, available treatments and most of the recommended first aid measures. Western medical treatment was preferred by the vast majority of the farmers over the traditional treatment.

**Conclusion:**

Some of the protective measures that the farmers were aware of are not practiced generally in Sri Lanka, suggesting a knowledge-practice mismatch. We suggest studies to understand the effects of socioeconomic and cultural determinants on snakebite prevention in Sri Lanka.

## Findings

### Introduction

The annual number of snakebites around the globe is estimated to be around 1.2-5.5 million. Of this, 81-95% occur in tropical regions of South Asia, South-East Asia, Sub- Saharan Africa and Latin America [1]. Large numbers of victims survive with permanent physical and psychological sequale, grossly affecting the ability to work and quality of remaining life [2]. Despite having this high disease burden [1], snakebite is still a neglected topic in the global health agenda. In Sri Lanka, around 37,000 snakebites are reported annually [3]. Of these, most bites are reported from the dry zone of Sri Lanka where they are among the three leading causes of admission to emergency care units at high prevalence districts [3]. As observed in other countries [4], snakebite is primarily an occupational hazard in Sri Lanka, involving farming communities [5]. Despite the fact that snakebite has been identified as a leading public health problem in the dry zones of the country, public health measures have failed to reduce the burden of this condition. Previous work has shown that snakebite and its complications could be avoided by educating the population at risk [6]. Specific measures related to housekeeping, outdoor work, healthcare seeking and home health practices have been identified as determinants of primary and secondary prevention of snakebite envenoming [6]. These measures are to be adopted by the population at risk; however, there should be targeted programs to educate people on these aspects. Studying the awareness and perceptions related to such issues of risk groups is important in designing community based interventional programs on snakebite towards combating the global burden of snakebite. Such information is lacking on the farming community of Sri Lanka. Hence, we studied the awareness, perceptions and treatment seeking behaviours related to snakebites among farmers in three districts in the dry zone of Sri Lanka.

### Methods

The study was conducted with the participation of 176 part-time and full-time, Chena and paddy farmers from Anuradhapura, Vavuniya and Jaffna districts of Sri Lanka, during July 2011 – November 2011. In all three districts, agriculture is the main economic activity. Snakebite has been reported as a leading public health problem in all study districts. Anuradhapura is the district with the highest burden of snakebite in Sri Lanka. Convenience sampling was adopted for this study and farmers were approached through farming organizations. An investigator assisted self completed questionnaire in Singhalese and Tamil languages was used for data collection. The validity of the translation was independently assessed by two observers competent in both languages. Relevant demographic data, awareness and perceptions on the venomous snakes in the area, first aid practices for snakebite, snakebite prevention and treatment were assessed via the questionnaire. The conduct of the study was approved by the Ethics Review Committee, Rajarata University of Sri Lanka. Verbal consent was sought from all participants prior to the participation.

### Results and Discussion

Participants of this study included 100 males (56.8%) and 76 females (43.2%), in the age range 16 - 70 years (mean 42 years, SD: 11 years), from Anuradhapura (n = 109), Jaffna (n = 45) and Vavuniya (n = 22) districts of Sri Lanka. The highest school education level of the participants was below grade 10 (26.7%), grade 10 - 12 (33.5%) and above grade 12 (39.8%). Both full time farmers (60.2%) and part time farmers (39.8%) were among the study participants. Interestingly, 30 farmers (17%) had a previous history of snakebite showing the magnitude of the burden of snakebite for farmers in this area. Table 1 describes the awareness and perceptions assessed on various aspects related to snakebites. A high percentage of the participants were aware of most of the recommended first aid measures. However, it was evident that a very high percentage of participants prefer the application of a tourniquet as a first aid measure following snakebite. This practice, although considered a dangerous first aid measure [7], has been observed among nearly 50% of viper bite victims in Sri Lanka [8].

**Table 1:** Awareness and perceptions of the 176 participant farmers on snakes, first aid, treatment and prevention of snakebites

**Awareness/perception**	**Number of participants responded**	**Yes**	**No**
***Venomous Snakes***			
Most of the snakes in Sri Lanka are non-venomous	176	131 (74.4%)	45 (25.6%)
***First aid***			
Bitten part of the body should be kept immobilized	171	153 (89.5%)	18 (10.5%)
Bitten site should not be excised	173	138 (79.8%)	35 (20.2%)
Aspirin should not be given for pain relief	169	131 (77.5%)	38 (22.5%)
Beverages containing alcohol should not be given to the patient for pain relief	172	153 (89.0%)	19 (11.0%)
Application of tight band (tourniquet) proximal to the site of bite	171	128 (74.9%)	43 (25.1%)
***Treatment***			
Capturing of the offending snake for identification is not essential in treating the patient	175	62 (35.4%)	113 (64.6%)
Snakebites can be successfully treated in Sri Lanka	169	126 (74.6%)	43 (25.4%)
Antivenom is available only in some hospitals in Sri Lanka	169	148 (87.6%)	21 (12.4%)
***Preventive measures***			
Avoiding of storing paddy harvest inside houses	172	143 (83.1%)	29 (16.9%)
Controlling rodents inside the houses	175	173 (98.9%)	2 (1.1%)
Storing firewood outside the houses	165	125 (75.8%)	40 (24.2%)
Clearing an area, devoid of leaf litter and grass around the houses	174	171 (98.3%)	3 (1.7%)
Tapping the ground with a stick, while walking outside at dusk	168	142 (84.5%)	26 (15.5%)
Carring a torch or a flame while walking outside at dusk	176	175 (99.4%)	1 (0.6%)
Wearing protective shoes while walking outside at dusk and while farming activities	171	160 (93.6%)	11 (6.4%)
***Preferred treatment method***			
Native/Ayurveda treatment		20 (11.5%)	
Western treatment from a government hospital		151 (86.8%)	
No special preference for one treatment method		3 (1.7%)	

Most participants believed the fact that snakebites could be successfully treated and were aware that snake antivenom is available in some hospitals in Sri Lanka. However, it is noteworthy that two thirds of the participants believed that capturing the snake for identification is essential for treating the victim. The only available antivenom in Sri Lanka is a polyvalent antivenom (Indian polyvalent antivenom), and the initiation of antivenom treatment is being decided based on the clinical evidences of envenoming and evidence for presence of a coagulopathy. Physical identification of the offending snake certainly would assist the physician in clinical decision making in treating snakebite victims. Hence, making the offending snake specimen available for medical staff for identification should be encouraged. However, non-availability of the snake for identification would not drastically alter the routine management of snakebite victims in Sri Lanka. Therefore, delays in taking the victim to medical care must be discouraged as life saving time would be lost. It is essential to communicate this message correctly to the communities at risk of snakebite.

The vast majority of the study participants preferred allopathic treatment for snakebites over traditional/Ayurveda treatment. Of these, 80.8% stated that the reason for their preference was the availability of government hospital within reach. High percentages of the participants were aware of the practices that minimize snakebites in houses and outdoors. Due to a lack of storing facilities, many small scale farmers in Sri Lanka tend to store paddy harvest within their houses. This could attract rodents and their predators (snakes) to houses. Although the vast majority of the participants were aware of this, it is uncertain that awareness will lead to a change in practice, unless practical solutions for harvest storage problems are provided for farmers.

Although 93.6% of the farmers were aware that wearing protective footwear would protect them against snakebite, it is highly unlikely that such measures would be adopted even by farmers who can easily afford protective footwear in Sri Lanka, because farming activities in Sri Lanka are almost always being conducted barefoot, due to the prevalent attitude of considering footwear as a burden. This study shows a high awareness of important preventive measures, first aid measures and available treatment for snakebites, among participant farmers in the three dry-zone districts. These figures on high awareness, however, do not reflect from the large number of hospital admissions due to snakebites and associated morbidity and mortality in the dry zone of Sri Lanka [3]. Therefore, this study suggests a possible knowledge-practice mismatch on snakebite prevention. Unfortunately, the influence of socio-economic and behavioral factors on snakebites has never been subjected to detailed study in Sri Lanka leaving little opportunity to understand the possible contributory factors. High awareness of snakebite prevention and first aid presumably is due to the fact that snakebite is an important part of the life of the studied population. Furthermore, the low priority given for snakebite prevention in community health promotion programs in Sri Lanka has presumably played a role in not bringing the knowledge into practice. However, unless the important knowledge gaps in the socioepidemiology of snakebites are filled and permanent snakebite prevention programs established, chances of changing practices towards minimizing snakebite appear slim in Sri Lanka.

## Competing interests

The authors declare that they have no competing interests.

## Authors’ contributions

AS conceived the study; AS, SA and FM planned the study; AS, FM, AM collected and AS and FM analyzed the data. AS drafted and SA critically reviewed the manuscript. All authors read and approved the final manuscript.
